# Colonic Microbiota Improves Fiber Digestion Ability and Enhances Absorption of Short-Chain Fatty Acids in Local Pigs of Hainan

**DOI:** 10.3390/microorganisms12061033

**Published:** 2024-05-21

**Authors:** Pengxiang Xue, Mingming Xue, Yabiao Luo, Qiguo Tang, Feng Wang, Ruiping Sun, Yanxia Song, Zhe Chao, Meiying Fang

**Affiliations:** 1 National Engineering Laboratory for Animal Breeding, MOA Laboratory of Animal Genetics and Breeding, Department of Animal Genetics and Breeding, College of Animal Science and Technology, China Agricultural University, Beijing 100193, China; xuepengxiang98@163.com (P.X.); mingmxue@163.com (M.X.); yabiaoluo2021@163.com (Y.L.); qgtango@163.com (Q.T.); 2 Institute of Animal Science and Veterinary Medicine, Hainan Academy of Agricultural Science, Haikou 571101, China; wfeng73cn@sina.com (F.W.); ruiping937@126.com (R.S.); chaozhe@hnaas.org.cn (Z.C.); 3 Sanya Institute, China Agricultural University, Sanya 572024, China; songyanxia2023@126.com

**Keywords:** Hainan pigs, dietary fiber, metagenome, metabolome, microbiota, SCFAs

## Abstract

Compared to commercial breeds, Chinese local pig breeds have a greater ability to digest dietary fiber, which may be due to differences in intestinal microbiota. In this study, we fed Ding’an and DLY pigs high and low levels of dietary fiber, respectively, to investigate factors contributing to high dietary fiber adaption in Ding’an pigs. Twelve Ding’an pigs and DLY pigs were randomly divided into a 2 (diet) × 2 (breed) factorial experiment (*n* = 3). Compared with commercial pigs, Ding’an pigs have a stronger ability to digest dietary fiber. *Prevotella* was more prevalent in Ding’an pigs than in DLY pigs, which may be an important reason for the stronger ability of fiber degradation in Ding’an pigs. When the effects of feed and breed factors are considered, differences in abundance of 31 species and 14 species, respectively, may result in a greater ability of fiber degradation in Ding’an pigs. Among them, *Prevotella.* sp. *CAG:520* may be a newly discovered bacterium related to fiber degradation, which positively correlated with many fiber-degrading bacteria (r > 0.7). We also found that the concentration of plant metabolites with anti-inflammatory and antioxidant effects was higher in the colonic chyme of Ding’an pigs after increasing the fiber content, which resulted in the downregulated expression of inflammatory factors in colonic mucosa. Spearman’s correlation coefficient revealed a strong correlation between microbiota and the apparent digestibility of dietary fiber (r > 0.7). The mRNA expressions of *SLC16A1*, *PYY*, and *GCG* were significantly increased in the colonic mucosa of Ding’an pigs fed on high-fiber diets, which indicates that Ding’an pigs have an enhanced absorption of SCFAs. Our results suggested that an appropriate increase in dietary fiber content can reduce the inflammatory response and improve feed efficiency in Ding’an pigs, and differences in the intestinal microbial composition may be an important reason for the difference in the fiber degradation capacity between the two breeds of pigs.

## 1. Background

The intestinal health of pigs can be improved in pig production by adding the proper amount of dietary fiber to the feed [1]. According to its solubility, dietary fiber can be divided into soluble dietary fiber (SDF) and insoluble dietary fiber (IDF), and IDF is more difficult to be utilized by the host [2]. The addition of appropriate dietary fiber to porcine diets has been shown to increase the α-diversity of gut microbes and induce alterations in β-diversity. These changes may potentially contribute to improved growth performance and enhanced resilience of the porcine gut microbiota [3]. Previous studies have demonstrated variations in the composition of gut microbiota among pigs with different feed efficiency, notably showing a significant increase in the abundance of microorganisms such as *Bifidobacterium*, *Clostridium*, and *Lactobacillus* in the pigs with higher feed efficiency [4]. The metabolites produced by these altered microorganisms may enhance host feed efficiency by influencing host gene expression or mitigating the host inflammatory response [5].

As a mammal, the pig cannot secrete cellulase by itself, and its utilization of cellulose is entirely dependent on the fermentation by a large number of microorganisms in the hindgut [6,7]. Dietary fiber is mainly fermented to produce short-chain fatty acids (SCFAs), which increase the weight of the hindgut and provide pigs with energy for daily activities and growth [8,9]. The interaction between fiber and intestinal microorganisms is mutual, the composition of microbiota determines the utilization of dietary fiber by the host, and in turn SCFAs can promote the growth of beneficial microorganisms such as cellulase-secreting bacteria and inhibit the growth of harmful microorganisms, thus changing the structure of the intestinal microbiota [10,11,12]. In addition, SCFAs can maintain glucose homeostasis in the host and promote the expression of tight junction proteins to enhance intestinal barrier function [13,14]. Previous studies have shown that the addition of different levels of dietary fiber to feed can alter the composition of the pig intestinal microbiota, and that intestinal microbiota differs between breeds of pigs [12,15,16]. However, excessive dietary fiber may reduce the digestibility of other nutrients by inhibiting the expression of some proteases, ultimately leading to lower growth performance in pigs [17,18].

Due to differences in the composition of the intestinal microbiota, Chinese local pigs may have a great ability to digest dietary fiber compared to commercial pigs [15,16,19]. A well-known and representative native breed in Hainan, the Ding’an pig, can accurately represent the characteristics of the Hainan pigs. There have been some studies on the roughage resistance of Ding’an pigs, but they have mainly focused on the collection of phenotypes, and so far there is a lack of research on the intestinal microorganisms of Ding’an pigs. The aim of this study was to investigate the impact of different levels of dietary fiber on intestinal microbial composition and metabolite production in Ding’an and DLY pigs by feeding them different proportions of alfalfa diets, while also identifying factors contributing to the greater adaptation of Ding’an pigs to higher fiber diets. We hypothesized that gut microbial composition varies in different breeds of pigs, which may contribute to differences in host utilization of dietary fiber.

## 2. Methods

### 2.1. Experimental Design and Sample Collection

Our study employed a prospective, randomized blinded experimental design, with pigs randomly assigned to different groups. We selected pigs from various pens, with one pig chosen from each pen based on the criteria of similar weight and absence of medical history. Twelve 60-day-old Ding’an pigs and DLY pigs (females) with average initial body weight of approximately 14.3 kg were randomly divided into high- and low-dietary fiber groups. In other words, the trial consisted of 4 groups with 3 replicates each: Ding’an pigs fed with high dietary fiber (DAH group), Ding’an pigs fed with low dietary fiber (DAL group), DLY pigs fed with high dietary fiber (DLYH group), and DLY pigs fed with low dietary fiber (DLYL group). All pigs were fed a corn–soybean diet, which complied with the nutritional requirements recommended by NRC (2020), prior to the trial. During the 56-day trial period, the high- and low-dietary fiber groups were given diets containing 10% and 5% dietary fiber (with some of the corn replaced by alfalfa), respectively. All experimental pigs were raised and managed in the same condition, and the animals were allowed to feed and drink freely during the experiment. The constituent components and nutrient composition of feed are listed in Table 1. All the pigs were slaughtered at the end of the trial, the dressing percentage was calculated, and feces samples were taken to determine the apparent digestibility of dietary fiber. Colonic contents were collected for metagenomic sequencing and metabolomic analysis. All samples were stored at −80 °C prior to sequencing.

### 2.2. Intestinal Morphology

The samples of foregut (duodenum, jejunum, and ileum) fixed in 4% paraformaldehyde solution and embedded in paraffin. Then, the samples were sectioned and stained with hematoxylin and eosin (H&E) by standard procedures. Images were captured at 200× magnification using Nikon Ti2-E. ImageJ software (Version: 1.53) was used to measure the villus height and crypt depth of the samples, and the ratio of villus height to crypt depth (V/C) was calculated.

### 2.3. Metagenomic Sequencing and Data Processing

Each sample was analyzed separately for metagenomic analysis. Total genomic DNA in the colonic content samples was extracted using Omega E.Z.N.A. Stool DNA Kit (Omega Bio-tek, Inc., Norcross, GA, USA) following the manual. The qualified DNA samples were sheared to 300 bp with the Covaris ultrasonic crusher and prepared into sequencing libraries after a series of processing. The metagenomic libraries were sequenced on Illumina Novaseq PE150 platform at Allwegene Technology Co., Ltd. (Beijing, China).

For the quality control of raw data, adapter sequence and low-quality reads were removed by using fastp [20]. The clean reads were mapped to the pig reference genome using BWA to filter out the host-origin reads [21], and the remaining reads were assembled to contigs using MEGAHIT [22]. The contigs were used for the prediction of open reading frames (ORFs) by using Prodigal, and ORFs over 100 bp were retrieved and translated into amino acid sequences using the NCBI translation table [23]. A non-redundant gene catalog with 90% sequence identity and 90% coverage was created. CD-HIT was used to construct a non-redundant gene catalog with 95% sequence identity and 90% coverage [24]. Clean reads were mapped to the non-redundant gene catalog with 95% identity using bowtie2, and gene abundance in each sample was evaluated [25]. Amino acid sequences were aligned with the NCBI NR database using DIAMOND, and species annotations were obtained from the taxonomic information database [26]. The KEGG annotation was conducted using DIAMOND against the KEGG database.

### 2.4. Metabolite Extraction, LC-MS Analysis, and Data Processing

The samples of colonic content (0.5 g) were mixed with 1.5 mL of H_2_O. The sample was centrifuged, and 100 μL of supernatant was mixed with 400 μL of extraction solution (Methanol/Acetonitrile = 1:1). Ultrasonication was performed for 10 min and then an hour of incubation at −20 °C. The samples were centrifuged at 4 °C and 13,400× *g* for 15 min, and then, 350 μL of supernatant was mixed with 150 μL of extraction solution. After ultrasonication in the ice water bath for 10 min, the samples were centrifuged at 4 °C and 13,400× *g* for 15 min, and the supernatant was ready for metabolite analysis.

Ultra-High-Performance Liquid Chromatography (UHPLC) separation was performed using an Acquity UHPLC system (Acquity LC, Waters, Milford, MA, USA) installed with a Waters UPLC column (ACQUITY UPLC BEH Amide, 1.7 µm, 2.1 × 100 mm). The UHPLC system was coupled with a Q Exactive mass spectrometer (Thermo Fisher Scientific, Waltham, MA, USA). Every program setting was applied in accordance with system requirements.

When a metabolite feature is found in fewer than 20% of experimental samples or in fewer than 50% of QC (quality control) samples, it is excluded from data analysis. Then, the missing values of raw data were filled up by half of the minimum value. Features with RSD > 30% should be removed from the subsequent analysis. Finally, 1735 metabolites were obtained. In order to obtain better group separation, metaX was used to perform orthogonal partial least squares discriminant analysis (OPLS-DA) [27]. To check the robustness and predictive ability of the OPLS-DA model, 200 permutations were further conducted to obtain the R2 and Q2 intercept values. The first principal component of variable importance in the projection (VIP) was obtained to evaluate the contribution of each variable to the model. The metabolites with VIP > 1, *p* < 0.05 (Student’s *t*-test), and fold change <0.67 or >1.5 were considered as differentially expressed metabolites. In addition, the pathways of metabolites were searched for using MetaboAnalyst and KEGG databases.

### 2.5. Bioinformatics Analysis

Linear discriminate analysis effect size (LEfSe) was performed using the online site Galaxy (http://huttenhower.sph.harvard.edu/galaxy/, accessed on 31 October 2022), with a set threshold of LDA > 2.0 and *p* < 0.05. The heat map analysis utilized R package heatmap. Spearman’s rank correlation coefficient and *p*-value were calculated to construct co-occurrence networks by R package according to the relative abundance distribution of the species. A connection represents a strong (r > 0.7 or r < −0.7) and significant (*p* < 0.01) correlation. Networks were constructed with Gephi (version 0.9.7).

### 2.6. RNA Isolation, Reverse Transcription, and Real-Time Quantitative PCR

Total RNA of colonic mucosa was isolated using Transzol reagent (TransGen Biotech Co., Ltd., Beijing, China) and then reverse transcribed into cDNA using the PrimeScript™ RT reagent Kit with gDNA Eraser (Takara Bio Inc., Dalian, China) according to the manufacturer’s protocol. Real-time quantitative PCR was performed using the TB Green^®^ Premix Ex Taq™ II Kit (Takara Bio Inc., Dalian, China) to detect the mRNA expression. The relative mRNA expression of the target genes was normalized to the housekeeping gene *GAPDH* or *β-actin* using the 2^−ΔΔCt^ method. The primer sequences are listed in Table S1.

### 2.7. Statistical Analysis

The data of phenotype in this research were analyzed using unpaired *t*-test or ANOVA procedure of the SAS 9.2 software (SAS Institute, Cary, NC, USA). Data are presented as mean ± SEM. GraphPad Prism8.0 (GraphPad Software, San Diego, CA, USA) was used to draw the histogram. Statistical significance is indicated as follows: * *p* < 0.05, ** *p* < 0.01, *** *p* < 0.001, and ns means no significance.

## 3. Results

### 3.1. Effect of Different Fiber Contents on Phenotype of Two Breeds of Pigs

**Figure 1:**
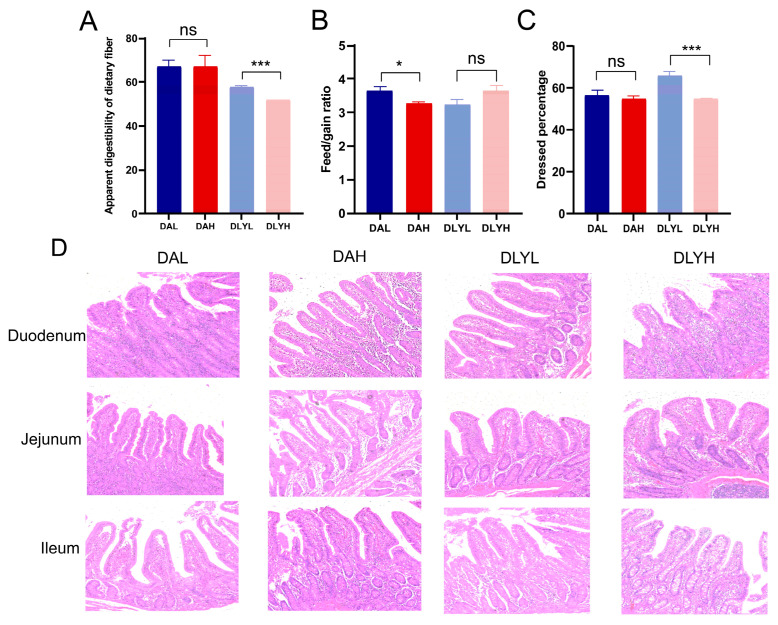
Effect of different fiber contents on the Ding’an pigs and DLY pigs. Changes in (**A**) apparent digestibility of dietary fiber, (**B**) feed/gain ratio, (**C**) dressed percentage, and (**D**) intestinal morphology (200×). DAH: Ding’an pigs fed with high dietary fiber; DAL: Ding’an pigs fed with low dietary fiber; DLYH: DLY pigs fed with high dietary fiber; and DLYL: DLY pigs fed with low dietary fiber. Data are mean ± SEM. * *p* < 0.05; *** *p* < 0.001; ns, no significance.

At the end of the experiment, we examined the apparent digestibility of dietary fiber and the feed/gain ratio of the four groups (Figure 1A,B). We found that increasing the dietary fiber concentration significantly decreased the apparent digestibility of dietary fiber in DLY pigs (*p* < 0.001), while it had no effect on Ding’an pigs. Additionally, after increasing dietary fiber, the feed/gain ratio of Ding’an pigs significantly reduced (*p* < 0.05), but DLY pigs displayed an increasing tendency. After the animals were slaughtered, we calculated the dressing percentage of all individuals (Figure 1C). We discovered that increasing dietary fiber had no effect on Ding’an pigs, while it significantly decreased the dressing percentage in DLY pigs (*p* < 0.001). These results suggest that, compared to DLY pigs, Ding’an pigs may digest more dietary fiber to provide energy for growth.

### 3.2. Influence of Dietary Fiber on Intestinal Morphology

The results of intestinal morphology of foregut, including the duodenum, jejunum, and ileum, are shown in Table 2 and Figure 1D. The villus height and V/C in the duodenum and ileum of DLY pigs were significantly reduced after increasing the dietary fiber content (*p* < 0.001), while those of Ding’an pigs were not affected. In both DLY pigs and Ding’an pigs, increasing the dietary fiber content had no effect on the crypt depth of the three intestinal segments.

### 3.3. Metagenomic Sequencing Data of Colonic Contents

Twelve samples from colonic contents of pigs were sequenced to explore the microbial composition, with an average of 85,222,539 raw reads per sample. The analysis showed that the rarefaction curves stabilized (Figure S1A). After removing low-quality sequences and host sequences, there were an average of 83,484,708 high-quality clean reads per sample. A total of 5,171,138 contigs were obtained after assembly, with an average of 430,928 contigs per sample. Finally, we obtained a non-redundant gene catalogue with 2,964,637 genes for further research, with an average length of 752 bp (Table S2).

### 3.4. Taxonomic Composition of Colonic Microbiota in the Four Groups

**Figure 2:**
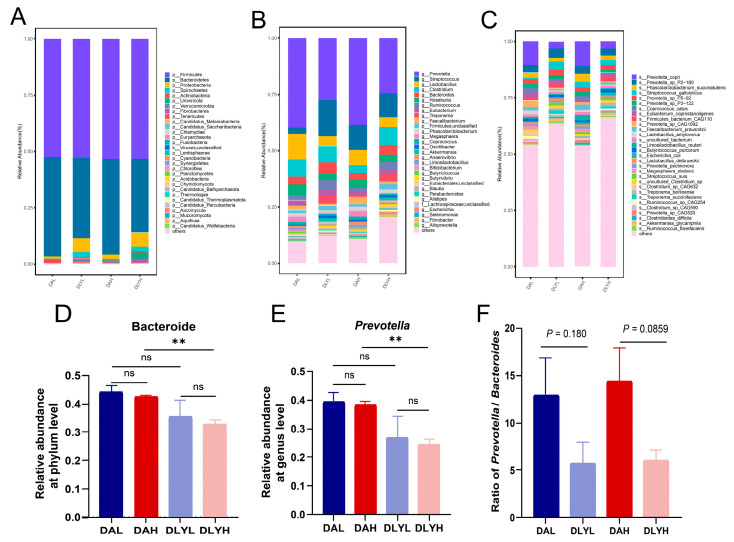
Composition of colonic microbiota in experimental pigs at different taxonomic levels by metagenomic sequencing. Relative abundance of dominant microbiota at the phylum (**A**), genus (**B**), and species (**C**) levels in the four groups is shown by stacked bar graphs. Relative abundance of (**D**) Bacteroide, (**E**) *Prevotella,* and (**F**) *Prevotella*/*Bacteroides* ratio. DAH: Ding’an pigs fed with high dietary fiber; DAL: Ding’an pigs fed with low dietary fiber; DLYH: DLY pigs fed with high dietary fiber; and DLYL: DLY pigs fed with low dietary fiber. Data are mean ± SEM. ** *p* < 0.01; ns, no significance.

The microbial composition of the colonic contents was discussed in order to understand the effects of low and high dietary fiber on the intestinal microbiota of these two breeds of pigs. The principal co-ordinates analysis (PCoA) revealed the differences in the composition of colonic microbiota between Ding’an pigs and DLY pigs (Figure S1B). At the phylum level, Firmicutes was most abundant in all four groups, followed by Bacteroidetes and Proteobacteria (Figure 2A). However, Bacteroidetes and Proteobacteria showed different trends in abundance in Ding’an pigs and DLY pigs. The abundance of Bacteroidetes in Ding’an pigs was higher than that in DLY pigs in both the low- and high-dietary fiber groups (Figure 2D, high dietary fiber: *p* = 0.0049, low dietary fiber: *p* = 0.239). In addition, the abundance of Spirochaetes was higher in DLY pigs than Ding’an pigs (high dietary fiber: *p* = 0.211, low dietary fiber: *p* = 0.0222). At the genus level, the dominant genera of all groups are *Prevotella*, *Streptococcus*, *Lactobacillus*, *Clostridium*, *Bacteroides*, *Roseburia*, *Ruminococcus*, *Eubacterium*, *Treponema,* and *Faecalibacterium* (Figure 2B). The abundance of *Prevotella* in Ding’an pigs was higher than that in DLY pigs in the high-dietary fiber groups (Figure 2E, high dietary fiber: *p* = 0.002, low dietary fiber: *p* = 0.198). We also calculated the ratio of *Prevotella* to *Bacteroides*, although it was not statistically significant, and the ratio was higher in Ding’an pigs (Figure 2F). We used LEfSe to identify biomarkers with significant differences between the four groups. Seven genera were significantly enriched in the DAH group, with *Limosilactobacillus* having the highest LDA score, which is a well-known probiotic. The largest number of biomarkers was found in the DLYH group, but most of these genera belong to pathogens (Figure S2A). Meanwhile, we found that half of the top ten species in terms of abundance belong to *Prevotella*, such as *Prevotella copri*, *Prevotella sp P2−180*, and *Prevotella sp P5−92* (Figure 2C). There were 20 biomarkers in the DAH group at the species level. Notably, species that have been reported to be positively correlated with dietary fiber addition were significantly enriched, such as *Eubacterium rectale*, *Blautia obeum*, and *Bacteroides acidifaciens*. Eighteen biomarkers were enriched in the DLYH group. In conformity with our expectations, most of these species were pathogenic microorganisms such as *Clostridium baratii*, *Enterobacter hormaechei*, *Clostridium perfringens*, and *Klebsiella variicola* (Figure S2B). In conclusion, there were significant differences in the colonic microbial composition among the four groups, especially between the two breeds of pigs.

### 3.5. Effect of Feed Factor on the Colonic Microbiota and Metabolites

**Figure 3:**
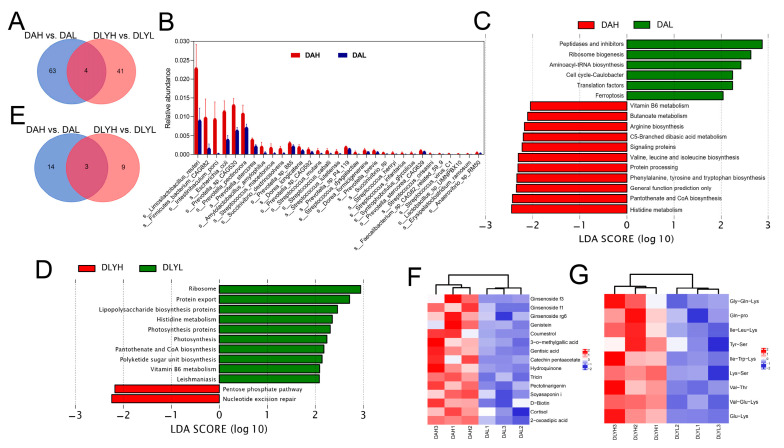
Effect of feed factor on the colonic microbiota and metabolites. (**A**) Venn diagram of the number of differential species. (**B**) Species with increased abundance specifically in Ding’an pigs after increasing fiber content. (**C**) Pathways with significant differential abundance between the DAH group and DAL group. (**D**) Pathways with significant differential abundance between the DLYH group and DLYL group. (**E**) Venn diagram of number of differential pathways. (**F**) Part of differential metabolites between DAH group and DAL group. (**G**) Part of differential metabolites between DLYH group and DLYL group. DAH: Ding’an pigs fed with high dietary fiber; DAL: Ding’an pigs fed with low dietary fiber; DLYH: DLY pigs fed with high dietary fiber; and DLYL: DLY pigs fed with low dietary fiber.

In order to understand the effect of dietary fiber on gut microbial composition, we conducted difference analysis on DAH vs. DAL group and DLYH vs. DLYL group. We found that there were differences in microbial composition between DAH and DAL groups (Figure S1B). The results showed that there were 67 differential species in DAH vs. DAL group and 45 differential species in DLYH vs. DLYL group (Figure S3). They share four differential species, which are *Bacteroides pectinophilus CAG437*, *Clostridium baratii*, *Clostridium* sp. *CAG465*, and *Clostridium* sp. *CAG678* (Figure 3A). There were 63 species specifically altered in Ding’an pigs, and 31 of which showed increased abundance after increasing dietary fiber content, such as *Limosilactobacillus reuteri*, *Firmicutes bacterium CAG882*, *Intestinibaculum porci*, and *Prevotella pectinovora* (Figure 3B). We speculated that the upregulation of the abundance of these species may allow Ding’an pigs to adapt to a higher dietary fiber content. Then, we compared the effects of different dietary fiber contents on intestinal microbial function. Seventeen differential pathways were enriched in Ding’an pigs, while twelve differential pathways were present in DLY pigs (Figure 3C,D). These two breeds of pigs shared three differential pathways, pantothenate and CoA biosyntheses, histidine metabolism, and vitamin B6 metabolism (Figure 3E). Interestingly, these three pathways showed completely contrary trends in the two breeds, with abundance of these pathways upregulated in Ding’an pigs and downregulated in DLY pigs after high dietary fiber feeding. To further understand the effect of different fiber contents on the metabolites of Ding’an pigs and DLY pigs, we performed untargeted metabolomics analysis of the colonic contents. The OPLS-DA model showed a separation of metabolite profiles for the four groups. According to the results of the substitution test, the model’s Q2 = 0.596 (*p* < 0.05) and R2Y = 0.991 (*p* < 0.05) values show that it has good explanatory and predictive power (Figure S4). Compared with the DAL group, we found that 2-oxoadipic acid and numerous plant metabolites, including ginsenoside, genistein, coumestrol, 3-o-methylgallic acid, gentisic acid, catechin pentaacetate, tricin, pectolinarigenin, and soyasaponin i were enriched in the DAH group (Figure 3F). Interestingly, compared with the DLYL group, we found a large number of dipeptides and tripeptides with increased abundance in the DLYH group (Figure 3G). Then, we examined the mRNA expression of several inflammatory factors, including *IFNG*, *IL-8*, *TNF-α*, *IL-18*, and *IL-26* (Figure S5). The mRNA expression of *IL-8* was significantly decreased in Ding’an pigs fed high dietary fiber, and the expression of other inflammatory factors also showed a decreasing trend.

### 3.6. Effect of Breed Factor on the Colonic Microbiota and Metabolites

**Figure 4:**
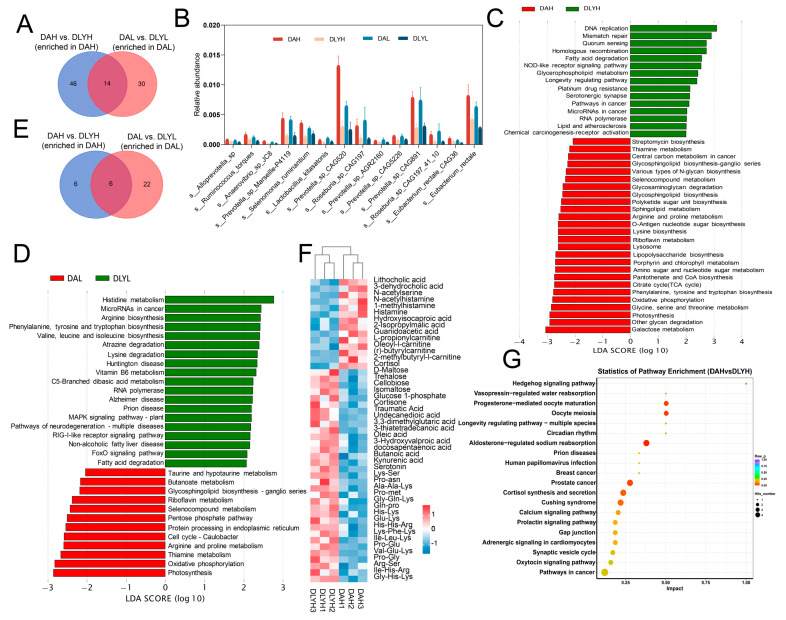
Effect of breed factor on the colonic microbiota and metabolites. (**A**) Venn diagram of the number of differential species. (**B**) Species with increased abundance in Ding’an both in high- and low-dietary fiber groups. (**C**) Pathways with significant differential abundance between the DAH group and DLYH group. (**D**) Pathways with significant differential abundance between the DAL group and DLYL group. (**E**) Venn diagram of number of differential pathways. (**F**) Part of differential metabolites between DAH group and DLYH group. (**G**) Pathway enrichment analysis was performed using the significantly different metabolites between DAH group and DLYH group. DAH: Ding’an pigs fed with high dietary fiber; DAL: Ding’an pigs fed with low dietary fiber; DLYH: DLY pigs fed with high dietary fiber; and DLYL: DLY pigs fed with low dietary fiber.

In order to investigate the differences in the composition and function of intestinal microorganisms among different breeds of pigs, we conducted difference analysis on DAH vs. DLYH group and DAL vs. DLYL group. The microbial composition of Ding’an pigs and DLY pigs was different in both the low-fiber and high-fiber diets (Figure S1B). There were 156 differential species in the DAH vs. DLYH group, and 146 differential species in the DAL vs. DLYL group (Figure S6). We found that fourteen species, both on high and low dietary fiber, were more abundant in Ding’an pigs than in DLY pigs (Figure 4A,B). We thought that these species are responsible for the higher apparent digestibility of dietary fiber in Ding’an pigs. We assessed the function of microorganisms by KEGG annotation. There were 41 and 31 differential functions in the high- and low-dietary fiber groups, respectively (Figure 4C,D). Notably, some amino acid metabolism pathways, such as histidine metabolism, arginine biosynthesis, and phenylalanine, tyrosine, and tryptophan biosynthesis, were significantly enriched in DLY pigs when fed on low dietary fiber. However, amino acid metabolism pathways such as glycine, serine, and threonine metabolism and lysine biosynthesis were represented in Ding’an pigs after increasing the dietary fiber content. Venn diagram showed the common enrichment pathways in Ding’an pigs in both comparisons (Figure 4E). Among them, thiamine metabolism, riboflavin metabolism, arginine and proline metabolism, and other metabolism pathways were significantly enriched. We displayed some metabolites that were specifically altered in the high-dietary fiber group by heatmap (i.e., not changed in low fiber feeding, Figure 4F). We found that several amino acid metabolites, including N-acetylserine, N-acetylhistamine, 1-methylhistamine, histamine, hydroxyisocaproic acid, 2-Isopropylmalic acid, and guanidoacetic acid, were enriched in the DAH group. In addition, two cholic acids and four l-carnitines were also found to be enriched in the DAH group. Eight fatty acids (docosahexaenoic acid, traumatic acid, undecanedioic acid, 3,3-dimethylglutaric acid, oleic acid, 3-hydroxyvalproic acid, docosapentaenoic acid, and butanoic acid) were enriched in the DLYH group. We also identified two tryptophan metabolites (kynurenic acid and serotonin) that were enriched in the DLYH group. For the differential metabolites, we further conducted pathway enrichment analysis, and we discovered that several metabolic pathways, including cortisol synthesis and secretion, starch and sucrose metabolism, nucleotide metabolism, and histidine metabolism, were enriched (Figure 4G). Notably, the metabolites enriched in histidine metabolism were all upregulated in the DAH group, suggesting that the pathway was activated in the DAH group.

### 3.7. Co-Occurrence Networks of Intestinal Microbiota

**Figure 5:**
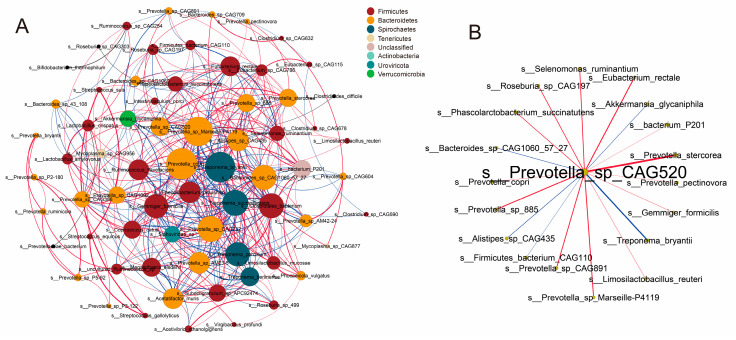
Co-occurrence networks of intestinal microbiota. (**A**) The co-occurrence networks among colonic microbiota in all samples. The nodes are colored by microbial phylum. The size of the node represents the weight of the species. (**B**) Species related to *P.* sp. *CAG:520*. The edges indicate correlations between nodes. Red and blue edges indicate positive and negative correlations, respectively. The thickness of the edges indicates the strength of the correlation.

The interaction between species in the colon was analyzed by co-occurrence network (Figure 5A). The figure only shows species that have interactions with more than two other species. We discovered that many of the differential bacteria identified with LEfSe were at the core of the co-occurrence network and interacted with many other bacteria, such as *I. porci*, *P.* sp. *CAG:520*, *S. ruminantium*, *E. rectale*, etc. Among the species screened for feed factor and breed factor, the *P.* sp. *CAG:520* was co-enriched. We found that *P.* sp. *CAG:520* was positively correlated with many fiber-degrading bacteria, such as *P. copri*, *E. rectale,* and *S. ruminantium* (Figure 5B).

### 3.8. Correlation Analysis of Phenotype, Microbiota, and Metabolites

**Figure 6:**
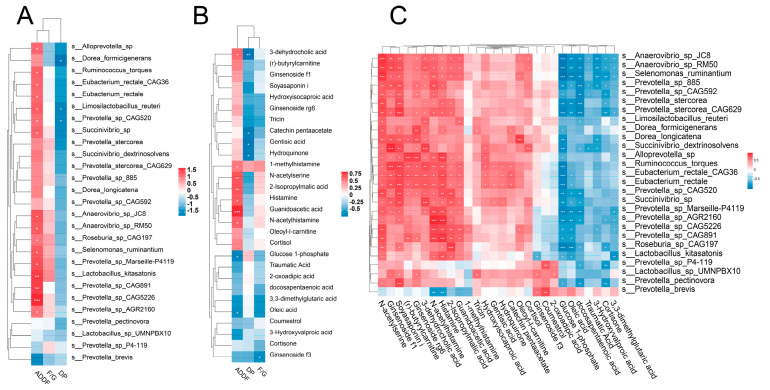
Correlation between the host phenotype, differential microbial species, and altered metabolites. Heatmap of Spearman’s correlation between (**A**) host phenotype and differential microbial species, (**B**) host phenotype and altered metabolites, and (**C**) differential microbial species and altered metabolites. ADDF, the apparent digestibility of dietary fiber; DP, dressed percentage; and F/G, feed/gain ratio. * *p* < 0.05; ** *p* < 0.01; *** *p* < 0.001.

We then analyzed correlations between important differential species, metabolites, and host phenotypes. We found a strong correlation between the apparent digestibility of dietary fiber and differential species or metabolites (Figure 6A,B). In agreement with our expectation, many fiber-degrading species such as *R. torques*, *E. rectale,* and *Prevotella* spp. were positively correlated with the apparent digestibility of dietary fiber. Strong correlation was also observed between these fiber-degrading species and metabolites (Figure 6C). Plant metabolites, including ginsenoside f1, soyasaponin i, ginsenoside rg6, etc., showed positive correlations with almost all of these species. Furthermore, amino acid metabolites, including N-acetylserine, N-acetylhistamine, 2-isopropylmalic acid, guanidinoacetic acid, etc., were also positively correlated with these species. Glucose 1-phosphate, oleic acid, and docosapentaenoic acid were negatively correlated with these species.

### 3.9. Increasing Fiber Content Can Improve the Absorption of SCFAs in Ding’an Pigs

**Figure 7:**
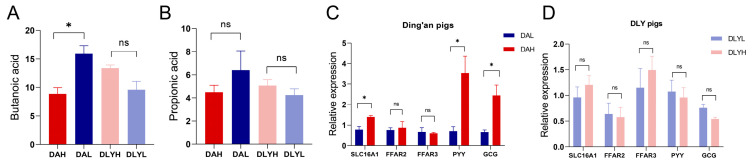
Relative concentrations of SCFAs and expression of genes related to SCFA sensing and absorption. Relative concentrations of (**A**) propionic acid and (**B**) butanoic acid. Changes in the expression of related genes in (**C**) Ding’an pigs and in (**D**) DLY pigs. *SLC16A*, monocarboxylate transporter 1; *FFAR*, free fatty acid receptor; *PYY*, peptide YY; and *GCG*, pro-glucagon. DAH: Ding’an pigs fed with high dietary fiber; DAL: Ding’an pigs fed with low dietary fiber; DLYH: DLY pigs fed with high dietary fiber; and DLYL: DLY pigs fed with low dietary fiber. Data are mean ± SEM. * *p* < 0.05; ns, no significance.

We detected the relative concentrations of two SCFAs, propionic acid and butanoic acid, through the untargeted metabolomics analysis (Figure 7A,B). After increasing dietary fiber, we found that the concentration of butanoic acid significantly decreased in Ding’an pigs (*p* < 0.05), and the concentration of propionic acid also showed a decreasing trend. Although the difference in DLY pigs was not statistically significant, both of these SCFAs showed an upward trend after increasing dietary fiber. However, we found that some well-known propionate- and butyrate-producing bacteria were most abundant in the DAH group (Figure S7). Then, we examined the mRNA expression of genes related to the absorption and sensing of SCFAs, including *SLC16A1*, *FFAR2*, *FFAR3*, *PYY*, and *GCG* (Figure 7C,D). The mRNA expression of *SLC16A1*, *PYY,* and *GCG* was significantly increased in Ding’an pigs fed high dietary fiber (*p* < 0.01). However, in DLY pigs, the dietary fiber content had no impact on the expression of these five genes.

## 4. Discussion

The purpose of this study was to explore the effects of low and high dietary fiber on Ding’an pigs and DLY pigs. In this study, we observed a significantly higher apparent digestibility of dietary fiber in Ding’an pigs compared to DLY pigs. Furthermore, we found a significant positive correlation between the apparent digestibility of dietary fiber and the abundance of several microorganisms enriched in Ding’an pigs. The enrichment of *Prevotella* in Ding’an pigs may contribute significantly to this difference. Additionally, we hypothesized that the increased production of SCFAs may stimulate the expression of specialized host-associated transporters and promote the uptake of SCFAs by the host.

The increase in the feed/gain ratio of DLY pigs fed high-fiber diets might be caused by the anti-nutritional factors in plant feeds, and adding excessive fiber may result in the decreased absorption of other nutrients [28]. In addition, alfalfa has high levels of insoluble dietary fiber, which may enhance the viscosity and speed up the transit of intestinal contents, thus reducing the contact time between digestive enzymes and intestinal chyme [29]. A possible reason why the performance of Ding’an pigs is not affected by increased fiber content is that they can degrade more dietary fiber to produce SCFAs to provide energy for host growth.

The colon of pigs contains a large number of microorganisms, and fiber is mainly digested in the colon, so we used colonic contents in the present study [30]. Previous studies have shown that the composition of gut microbes is regulated by the genetic factors of the host [31]. The results showed that Firmicutes and Bacteroidetes were the dominant phyla in all four groups, which is consistent with the previous studies [7,15]. Bacteroidetes are the most dominant phylum in the rumen of ruminants, and they can encode a large number of cellulases to digest dietary fiber, and we found that the proportion of Bacteroidetes in Ding’an pigs was higher than that in DLY pigs [32]. *Prevotella* is an important genus of the Bacteroidetes and has been shown to have a strong ability for digestion of dietary fiber [33]. There is a relationship between the ratio of *Prevotella* to *Bacteroides* and the utilization of dietary fiber [34,35]. High levels of *Prevotella* are associated with high-fiber diets, whereas high levels of *Bacteroides* are linked to high-protein diets. *Prevotella* spp. encode numerous polysaccharide-digesting enzymes, allowing them to efficiently produce propionate from arabinoxylans and cellulose [36]. A previous study had demonstrated that mice colonized with *P. copri*, a producer of succinate, can sustain host glucose homeostasis on a high-fiber diet [37]. However, this bacterium has been reported to cause chronic inflammation in the host through the activation of mTOR and TLR4 signaling pathway, ultimately leading to increased fat accumulation in pigs [38]. In conclusion, the composition of microbiota in the colon of Ding’an pigs is more suited to the digestion of dietary fiber. However, these bacteria also have the potential to contribute to the development of inflammation, which can lead to fat deposition in the host. This might be a significant reason for the low leanness of Chinese local pig breeds.

LEfSe is a method for finding differences between groups that takes into account both statistical significance and biological consistency [39]. At the genus level, there were seven biomarkers in the DAH group. *Limosilactobacillus* is a well-known prebiotic that protects the gut of host from pathogens [40]. *Selenomonas* is a genus frequently found in the rumen of ruminants, which is a producer of SCFAs [41]. However, the biomarkers in the DLYH group were all viruses or pathogenic bacteria. At the species level, we found similar findings, with enrichment in the DAH group for species related to dietary fiber utilization and SCFAs production, such as *E. rectale*, *B. obeum,* and *B. acidifaciens* [42,43,44]. And most of the microorganisms enriched in the DLYH group are pathogens [45,46]. In conclusion, we believed that the high apparent digestibility of dietary fiber of Ding’an pigs is associated with the numerous microorganisms exhibiting the function of dietary fiber degradation, such as *E. rectale*, *B. obeum*, etc. And we speculated that the increased level of pathogens in the intestinal tract of DLY pigs fed high-fiber diets impairs the health of the host intestine, resulting in decreased nutritional absorption.

We found 31 species that increased in Ding’an pigs only after being fed high-fiber diets when the effect of feed factor was considered. Although *L. reuteri* does not degrade dietary fiber, it can strengthen the immune system and intestinal barrier of the host [47]. *P. pectinovora* can produce acetate and a small amount of propionate by the fermentation of pectin [48]. *P. stercorea* can encode several carbohydrate esterases, which can promote the degradation of carbohydrate [49]. We hypothesize that the upregulation of the abundance of these species may have enabled Ding’an pigs to adapt to higher dietary fiber content. Three metabolic pathways showed opposite trends in the two breeds of pigs, which were histidine metabolism, pantothenate and CoA biosyntheses, and vitamin B6 metabolism. Coenzyme A (CoA) is a necessary cofactor for the metabolism of energy, and we think that microorganisms in the DAH group produce more CoA to help with the metabolism of dietary nutrients [50]. And vitamin B6 can improve immune function and reduce inflammation of the host [51]. The histidine metabolism pathway was enriched through both the metagenome and metabolome, and three of these metabolites in this pathway showed a positive correlation with the apparent digestibility of crude fiber, which may suggest that this pathway plays an important role. However, research on the precise mechanisms by which this route influences the targeted traits is still lacking. In addition, we found that butanoate metabolism was specifically enriched in the DAH group and that this pathway was associated with fiber degradation. We found 14 species that increased in Ding’an pigs compared to DLY pigs whether fed high- or low-fiber diets when the effect of breed factor was considered. The majority of these species are members of the genera *Prevotella*, *Roseburia*, and *Eubacterium*, which have been reported to be associated with dietary fiber utilization or feed efficiency [33,52,53]. In addition, *S. ruminantium* is a dominant bacterium in the rumen, which can promote fiber fermentation to produce propionate [54]. The relative abundance of *P.* sp. *CAG:520* was significantly different among the four groups and was positively correlated with many fiber-degrading bacteria. These results may indicate that this bacterium plays a key role in the digestion of fiber in Ding’an pigs. The enrichment of the glycan metabolism-related pathways in Ding’an pigs may indicate that intestinal bacteria in Ding’an pigs can utilize host epithelial cell glycans as a source of energy [55]. These results suggested that the colonic microbiota of Ding’an pigs can be altered to help hosts adapt to high-fiber diets and that minority of species may play a key role.

We discovered that increasing dietary fiber resulted in a significant increase in the abundance of plant metabolites with anti-inflammatory and antioxidant effects in Ding’an pigs [56,57,58]. As we described above, some bacteria in the colon of Ding’an pigs, such as *P. copri* and *Streptococcus* spp., may cause inflammation in the host. We suggested that these plant metabolites may lessen the harm caused by these bacteria to the intestinal barrier because the expression of inflammatory factors in the intestinal mucosa of Ding’an pigs decreased. Previous studies have shown that the addition of dietary fiber may decrease the digestibility of protein in the foregut [59]. Our research revealed that adding more dietary fiber to DLY pigs reduced the villus height in the foregut, which may have a negative impact on the dietary protein absorption. This observation aligns with previous research indicating that elevating dietary fiber levels leads to a reduction in the length of small intestinal villi [60]. It is hypothesized that the presence of anti-nutritional factors within the fibers could potentially contribute to villous atrophy. Therefore, we detected more dipeptides and tripeptides in the intestinal contents of DLY pigs fed high-fiber diets. We hypothesize that, as more proteins reach the colon of DLY pigs, the pH of their colon increases, which causes an increase in pathogens.

The concentration of SCFAs in colonic chyme is dynamic and is influenced by the microbial degradation of dietary fiber as well as the absorption by the host. More than 90% of SCFAs are rapidly absorbed in the colonic lumen [61]. Contrary to our expectations, the concentration of SCFAs in the colonic chyme of Ding’an pigs decreased after increasing the fiber content. SCFAs enter the colonic epithelial cells mainly through MCT1 encoded by *SLC16A1* [62]. *PYY* and *GCG* play important roles in the control of host insulin secretion and maintenance of glucose homeostasis [63]. Previous studies have demonstrated that the increased absorption of SCFAs by the host can promote the expression of *GCG* and *PYY* [64,65]. We proposed that a higher concentration of SCFAs produced by high-fiber diets stimulate the expression of *SLC16A1*, which improves the absorption of SCFAs in Ding’an pigs and ultimately results in higher expression of *PYY* and *GCG*. Previous studies have shown that SCFAs can reduce the inflammatory response of the intestinal mucosa [66]. Therefore, the increased absorption of SCFAs in Ding’an pigs may also be an important reason for the decreased expression of inflammatory factors in the colonic mucosa. The limitations of our study include a small sample size and the inability to isolate enriched *Prevotella* strains. Future studies should prioritize expanding sample sizes to enhance reliability and generalizability. Additionally, isolating specific *Prevotella* strains for in vitro investigations may provide valuable insights for practical applications. For example, in vitro fermentation and safety studies on selected *Prevotella* strains can be conducted as potential feed additives to improve fiber utilization in animals.

## 5. Conclusions

Compared with DLY pigs, Ding’an pigs have better digestibility of dietary fiber. We found that the colonic microbial composition of Ding’an pigs and DLY pigs were significantly different through metagenome. We also identified some important microorganisms that may be involved in the fiber degradation in Ding’an pigs, such as *P.* sp. *CAG:520*, *P. stercorea* and *P. copri*. Excessive dietary fiber can impair the small intestinal villi of DLY pigs, leading to the backward movement of dietary protein in the digestive tract, which promotes the growth of pathogens in the colon. Furthermore, our results provide a different perspective that increasing fiber does not necessarily increase the concentration of SCFAs in the intestine, because the concentration of SCFAs is also related to the absorption capacity of the host. In conclusion, the colonic microbiota of Ding’an pigs had a higher potential of fiber degradation and could adapt to changes in the dietary fiber content. In addition, the histidine metabolic pathway may play an important role. Further studies need to focus on the specific mechanisms underlying the effects of histidine metabolic pathways on the digestion process of dietary fiber.

## Figures and Tables

**Table 1:** Ingredients and nutrient levels of the experimental diets (as-fed basis).

Item	Dietary Fiber 5%	Dietary Fiber 10%
Ingredients%		
Corn	49.4	32.7
Alfalfa	2.7	16.9
Wheat bran	13.1	13.1
Soybean meal	18	18
Rice bran	13	13
Soybean oil	1	3.5
Limestone	1.4	1.4
Sodium chloride	0.3	0.3
L-Lysine	0.1	0.1
Premix	1	1
Total	100	100
Nutrient level *		
DE, MJ·kg^−1^	13	12.89
CP %	16.01	16.3
Lys %	0.85	0.84
Met + Cys %	0.44	0.43
Trp %	0.14	0.14
Thr %	0.54	0.53
Dietary fiber %	5	10
Calcium %	0.64	0.65
Phosphorus %	0.55	0.54

* DE was a calculated value, while the other nutrient levels were measured values.

**Table 2:** Effect of feeding two different dietary fiber contents (high and low) in Ding’an pigs (*n* = 3) and DLY pigs (*n* = 3).

Item	Ding’an Pigs		DLY Pigs		SEM	*p*-Value		
	DAL	DAH	DLYL	DLYH		Breed	DF	Interaction
Duodenum								
Villus height (μm)	377 ± 1 ^b^	374 ± 3 ^b^	416 ± 6 ^a^	375 ± 2 ^b^	1.636	0.001	0.001	0.001
Crypt depth (μm)	171 ± 5 ^b^	163 ± 5 ^b^	200 ± 6 ^a^	202 ± 6 ^a^	2.737	0.001	0.626	0.354
V/C	2.25 ± 0.06 ^ab^	2.34 ± 0.06 ^a^	2.11 ± 0.06 ^b^	1.92 ± 0.06 ^c^	0.030	0.001	0.378	0.019
Jejunum								
Villus height (μm)	412 ± 6 ^a^	397 ± 6 ^ab^	389 ± 6 ^b^	393 ± 4 ^ab^	2.815	0.020	0.359	0.098
Crypt depth (μm)	185 ± 8 ^a^	179 ± 5 ^a^	175 ± 4 ^a^	172 ± 5 ^a^	2.874	0.114	0.474	0.798
V/C	2.30 ± 0.08 ^a^	2.25 ± 0.06 ^a^	2.25 ± 0.04 ^a^	2.33 ± 0.07 ^a^	0.032	0.726	0.793	0.318
Ileum								
Villus height (μm)	393 ± 6 ^a^	405 ± 6 ^a^	398 ± 5 ^a^	353 ± 3 ^b^	2.602	0.001	0.002	0.001
Crypt depth (μm)	194 ± 6 ^a^	193 ± 6 ^a^	185 ± 5 ^a^	188 ± 6 ^a^	2.819	0.227	0.820	0.744
V/C	2.06 ± 0.05 ^ab^	2.13 ± 0.06 ^a^	2.18 ± 0.05 ^a^	1.92 ± 0.07 ^b^	0.029	0.451	0.129	0.006

^a,b,c^ Means with different superscripts in the same row differ significantly (*n* = 3, *p* < 0.05). V/C, the ratio of villus height to crypt depth. SEM, standard error of the mean.

## Data Availability

The datasets supporting the conclusions of this article are available in the NCBI BioProject database (accession number: PRJNA942432).

## Supplementary Materials

The following supporting information can be downloaded at: https://www.mdpi.com/article/10.3390/microorganisms12061033/s1, Figure S1: Rarefaction curves and β-diversity analysis; Figure S2: Identification of the biomarker for each group by LEfSe at (A) genus level and (B) species level; Figure S3: Differential species between (A) DAH group and DAL group, (B) DLYH group and DLYL group; Figure S4: Orthogonal projection to latent structure-discriminant analysis (OPLS-DA) of metabolite composition in the four groups; Figure S5: Expression of inflammatory factors in the colonic mucosa of (A) Ding’an pigs and (B) DLY pigs; Figure S6: Differential species between (A) DAH group and DLYH group, (B) DAL group and DLYL group; Figure S7: The relative abundance of propionate and butyrate-producing bacteria in the four groups; Table S1: Primer sequences used for quantitative real-time PCR; Table S2: Summary of metagenomic sequencing data.

## Abbreviations

DLY	Duroc × Landrace × Yorkshire
H&E	Hematoxylin–eosin
V/C	Villus height to crypt depth
PCoA	Principal co-ordinates analysis
LEfSe	Linear discriminate analysis effect size
LDA	Linear discriminant analysis
KEGG	Kyoto Encyclopedia of Genes and Genomes
OPLS-DA	Orthogonal partial least squares discriminant analysis
VIP	Variable importance in the projection
SEM	Standard error of mean
SCFAs	Short-chain fatty acids
