# Supplementary material for: Colonic Microbiota Improves Fiber Digestion Ability and Enhances Absorption of Short-Chain Fatty Acids in Local Pigs of Hainan
Source: Microorganisms. 2024 May 21;12(6):1033. doi: 10.3390/microorganisms12061033 (PMC11205767; doi:10.3390/microorganisms12061033)
Supplement: Supplementary file 1 [file microorganisms-12-01033-s001.zip › microorganisms-2965695-supplementary.pdf]

Colonic Microbiota Improves Fiber Digestion Ability and Enhances Absorption of SCFAs in Hainan Local Pigs

Pengxiang Xue[1*], Mingming Xue[1*], Yabiao Luo[1], Qiguo Tang[1], Feng Wang[2], Ruiping Sun[2], Zhe Chao[2], Meiying Fang[1,3#]

[1] Department of Animal Genetics and Breeding, National Engineering Laboratory for Animal Breeding, MOA Laboratory of Animal Genetics and Breeding, College of Animal Science and Technology, China Agricultural University, Beijing, China.

[2] Institute of Animal Science and Veterinary Medicine, Hainan Academy of Agricultural Science, Haikou, China

[3] Sanya Institute of China Agricultural University, Sanya, China.

#Address correspondence to Meiying Fang, meiying@cau.edu.cn

*Pengxiang Xue and Mingming Xue contributed equally to this work.

Table S1. Primer sequences used for quantitative real-time PCR

Gene symbol	Primer sequence (5′ - 3′)	Product size (bp)
GAPDH	F: ACAGTCAAGGCGGAGAACG<br>R: CATTTGATGTTGGCGGGAT	86
SLC16A1	F: TCATCTCAGGCATCTATCT<br>R: GCTTCTCAGCAGCGTCTAT	132
FFAR2	F: ACTTGAACTCAACCCAGAAGG<br>R: CATGATCCACACAAAGCGCGAGT	173
FFAR3	F: GCTCCTGTTGCTCTTCCTG<br>R: ACGTGAGATAGACGGTGGTG	122
PYY	F: GCCGCTACTACGCCTCCCT<br>R: AGCGTCTGGGCTGTCACGT	80
GCG	F: ATTCATTGCTTGGCTGGTG<br>R: GTCTGCGGCGGAGTTCTTC	83
β-actin	F: GGCACCACACCTTCTACAACGAG<br>R: TCATCTTCTCACGGTTGGCTTTGG	102
IFNG	F: TCCAGCGCAAAGCCATCAGTG<br>R: ATGCTCTCTGGCCTTGGAACATAGT	111
IL-8	F: GAAGAGAACTGAGAAGCAACAACA<br>R: TTGTGTTGGCATCTTTACTGAGA	99
TNF-α	F: CCACGCTCTTCTGCCTACTGC<br>R: GCTGTCCCTCGGCTTTGAC	168

IL-18	F: ACGATGAAGACCTGGAATCGG<br>R: AAACACGGCTTGATGTCCCT	113
IL-26	F: AGCGTGCAAGGAAACAGACT<br>R: CAGGAAATACAGCGGCTCA	72

SLC16A1, monocarboxylate transporter 1; *FFAR*, free fatty acid receptor; *PYY*, peptide YY; *GCG*, pro-glucagon; *IFNG*, interferon gamma; *IL-8*, interleukin-8; *TNF-α*, tumor necrosis factor-α; *IL-18*, interleukin-18; *IL-26*, interleukin-26.

Table S2. Summary of metagenomic sequencing data

Sample	Total reads	Raw bases(GB)	Clean reads	Number of contigs	Total length(bp)	Average length(bp)	N50(bp)
DAH1	84,724,416	12.1199	83,383,214	496,697	684,325,088	1,378	1,692
DAH2	84,877,128	12.1418	80,192,868	307,103	480,480,021	1,565	2,226
DAH3	83,292,452	11.9151	81,224,658	363,783	521,596,497	1,434	1,860
DAL1	84,649,036	12.1091	83,293,264	376,012	553,863,710	1,473	1,977
DAL2	84,650,040	12.1093	82,848,320	358,808	532,381,185	1,484	2,003
DAL3	83,411,334	11.9321	82,769,460	353,461	569,599,294	1,612	2,395
DLYH1	84,840,014	12.1365	84,287,132	543,636	711,334,672	1,309	1,525
DLYH2	84,417,818	12.0761	83,951,394	509,084	654,767,202	1,286	1,489
DLYH3	86,126,816	12.3205	84,862,008	548,800	738,711,724	1,346	1,608
DLYL1	89,342,660	12.7806	87,833,792	452,588	650,373,702	1,437	1,852
DLYL2	87,891,832	12.573	84,197,566	405,816	541,643,585	1,335	1,582
DLYL3	84,446,924	12.0802	82,972,822	455,350	664,363,126	1,459	1,911

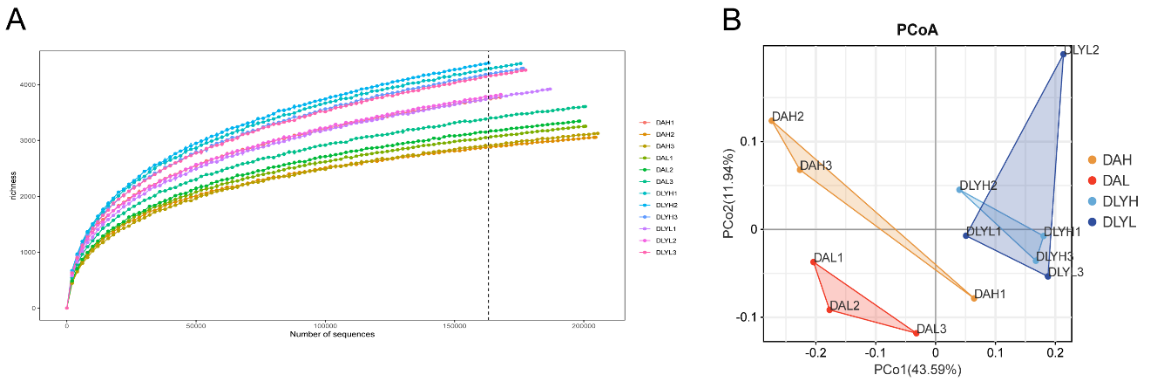

Figure S1. Rarefaction curves and β-diversity analysis. (A) a Rarefaction curves for richness in all samples. **(B)** Principal co-ordinates analysis (PCoA) based on Bray-Curtis distance.

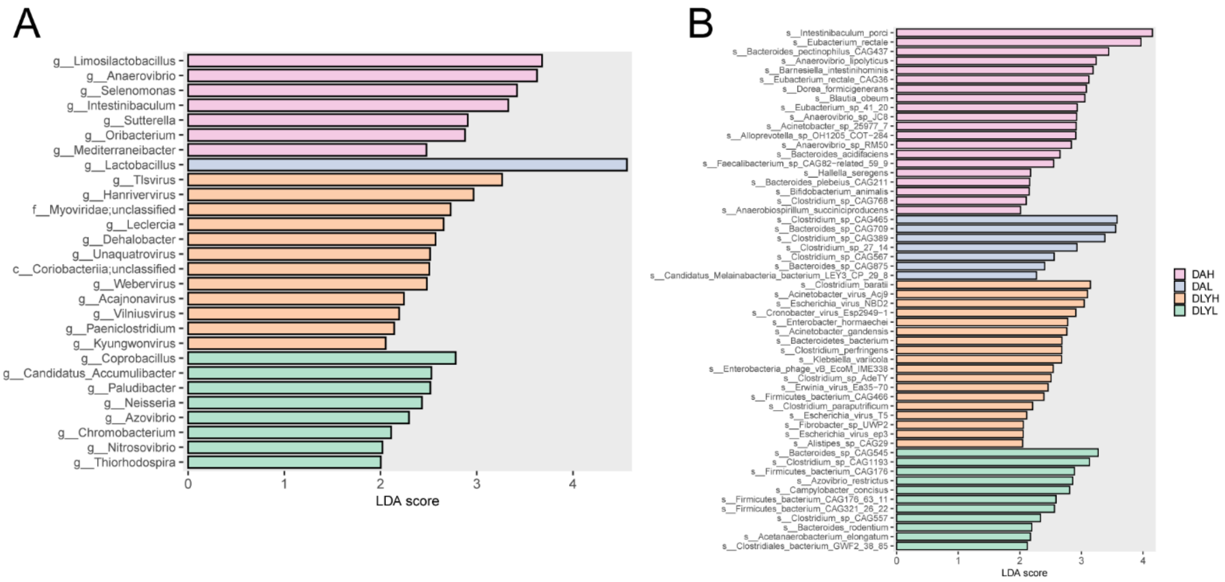

Figure S2. Identification of the biomarker for each group by LEfSe at **(A)** genus level and **(B)** species level.

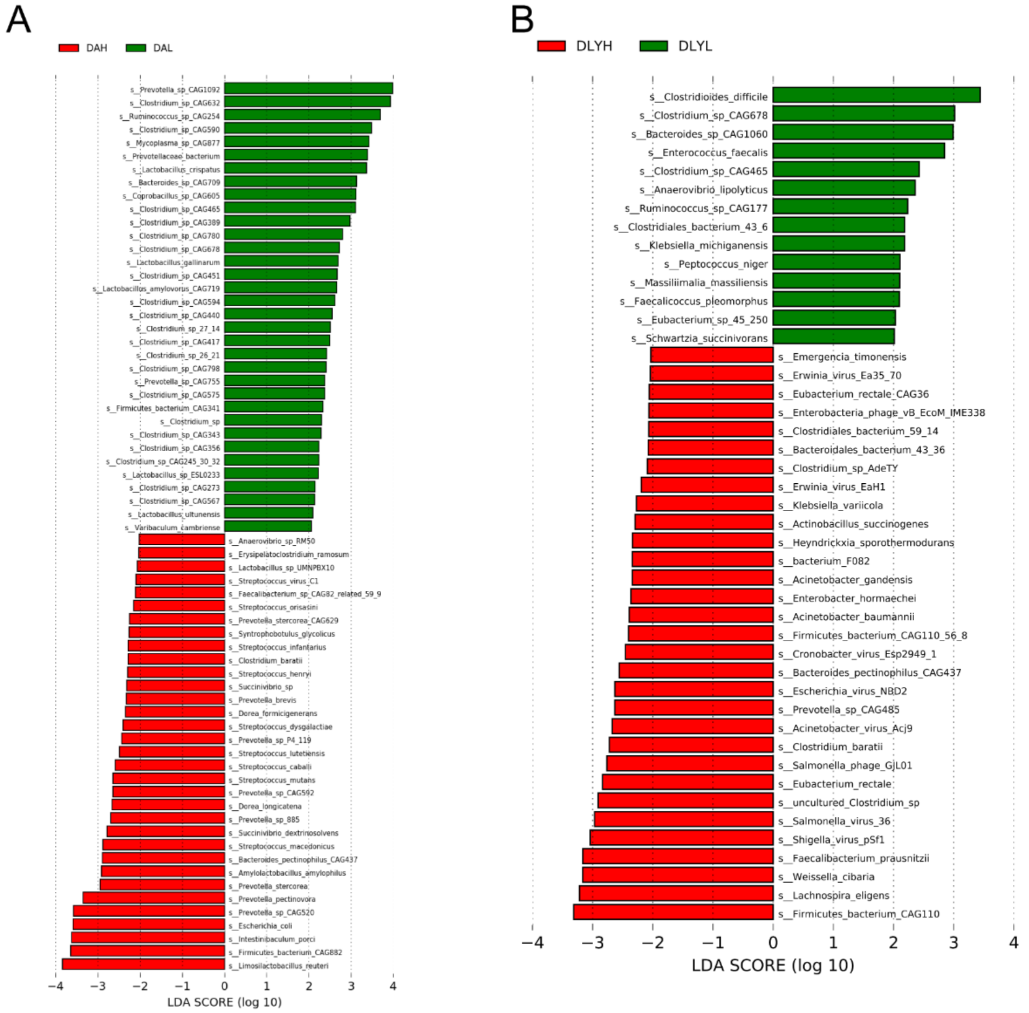

Figure S3. Differential species between **(A)** DAH group and DAL group, **(B)** DLYH group and DLYL group.

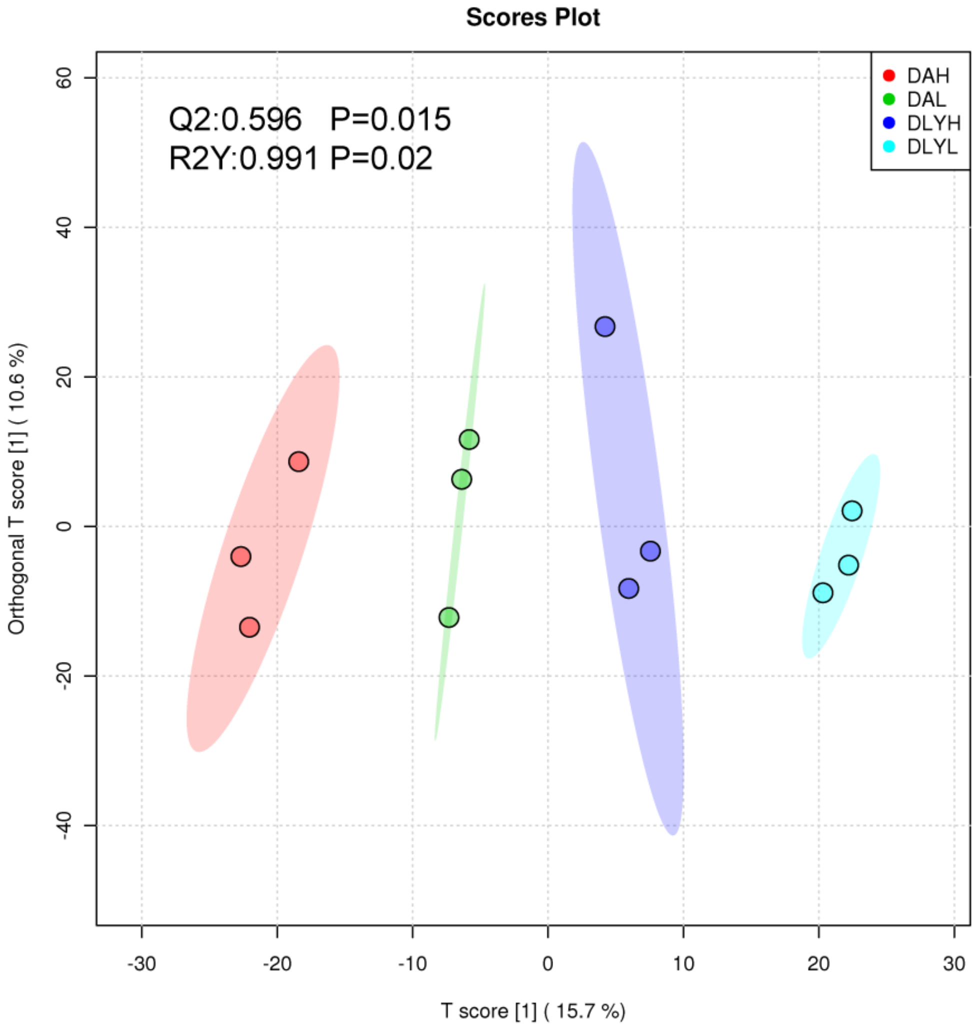

Figure S4. Orthogonal projection to latent structure-discriminant analysis (OPLS-DA) of metabolite composition in the four groups.

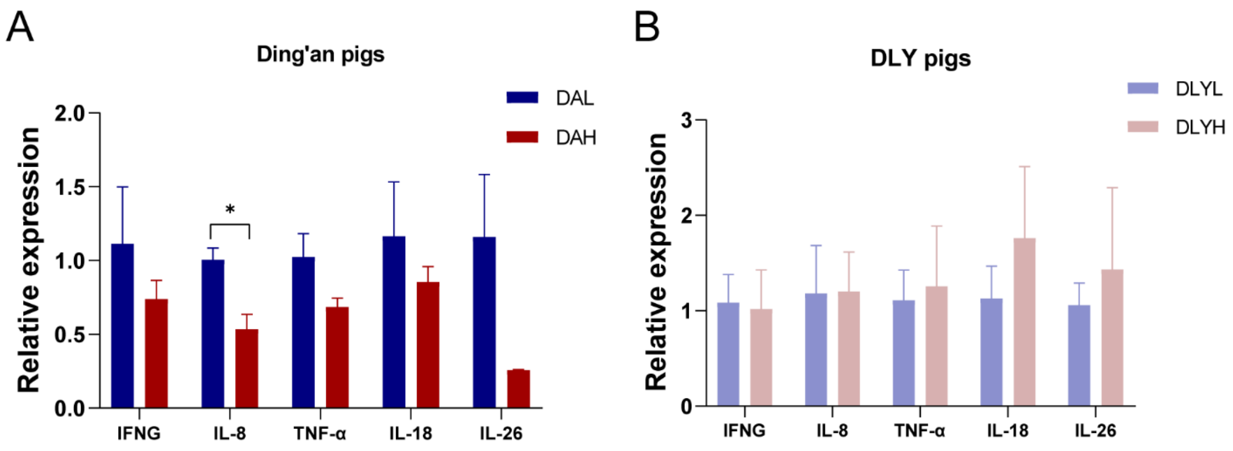

Figure S5. Expression of inflammatory factors in the colonic mucosa of **(A)** Ding'an pigs and **(B)** DLY pigs.

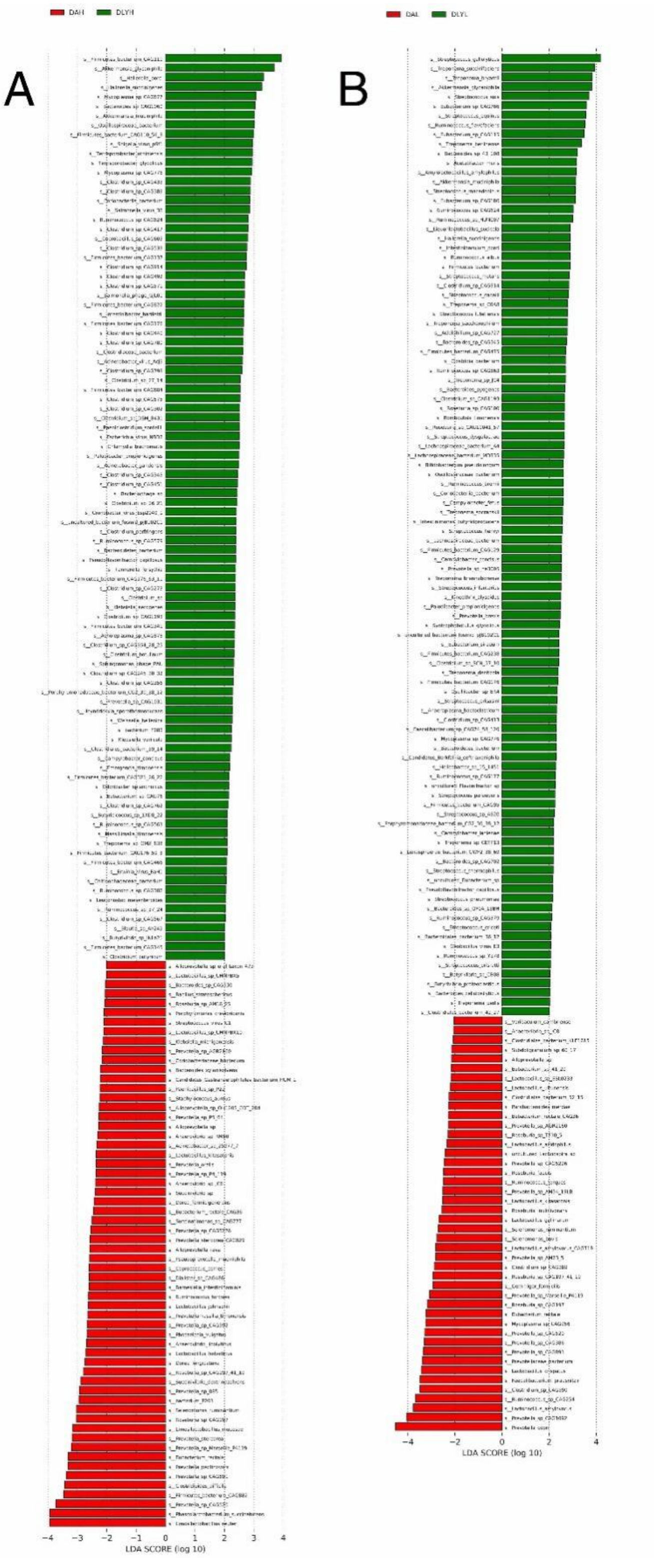

Figure S6. Differential species between **(A)** DAH group and DLYH group, **(B)** DAL group and DLYL group.

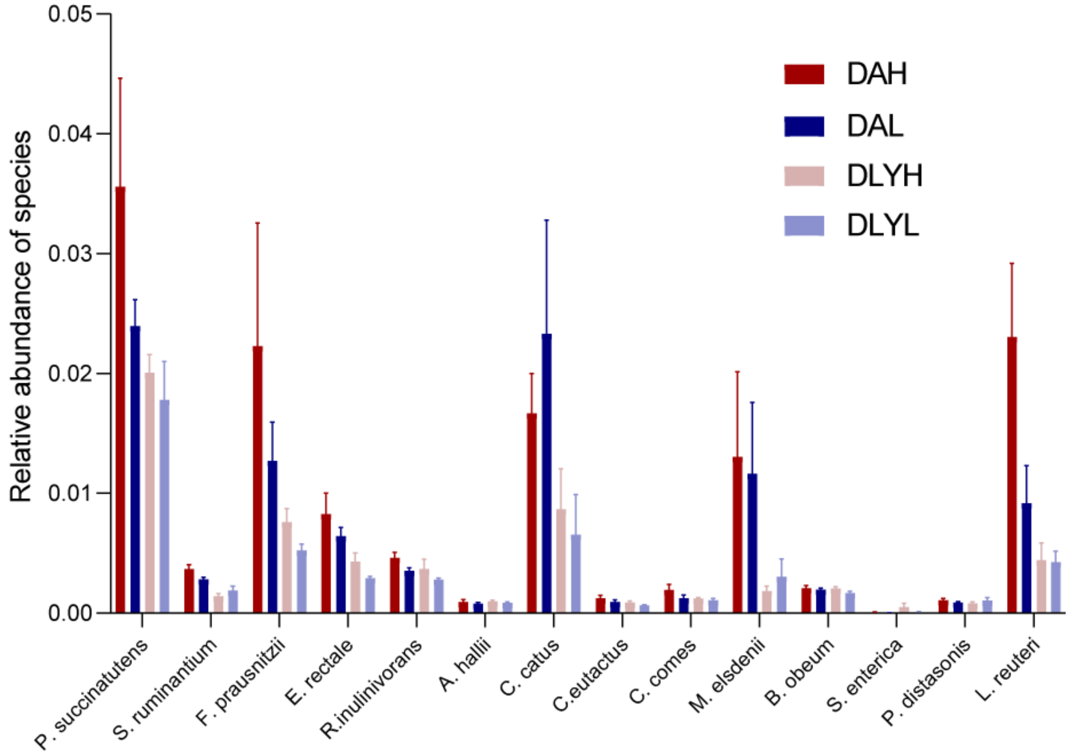

Figure S7. The relative abundance of propionate and butyrate-producing bacteria in the four groups.
